# Nutritional Status in Multiple Sclerosis, Neuromyelitis Optica Spectrum Disorder, and Myelin Oligodendrocyte Glycoprotein Antibody-Associated Disease

**DOI:** 10.14740/jocmr6676

**Published:** 2026-09-26

**Authors:** Ekdanai Uawithya, Teerawat Koosirirat, Natnasak Apiraksattayakul, Punchika Kosiyakul, Sasitorn Siritho, Tatchaporn Ongphichetmetha, Kusuma Chaiyasoot, Jerawat Uthankul, Natthapon Rattanathamsakul, Jiraporn Jitprapaikulsan

**Affiliations:** a Siriraj Neuroimmunology Center, Division of Neurology, Department of Medicine, Faculty of Medicine, Siriraj Hospital, Mahidol University, Bangkok, Thailand; b Department of Neurology, Oklahoma University Health Science Center, Oklahoma City, OK, USA; c Department of Neurology, Faculty of Medicine, Siriraj Hospital, Mahidol University, Bangkok, Thailand; d Department of Psychiatry, Texas Tech Health Science Center, El Paso, TX, USA; e Bumrungrad International Hospital, Bangkok, Thailand; f Division of Clinical Epidemiology, Department of Research and Development, Faculty of Medicine, Siriraj Hospital, Mahidol University, Bangkok, Thailand; g Division of Clinical Nutrition, Department of Medicine, Siriraj Hospital, Mahidol University, Bangkok, Thailand; h Faculty of Medicine, Siriraj Hospital, Mahidol University, Bangkok, Thailand

**Keywords:** MS, NMOSD, MOGAD, Malnutrition, Nutritional assessment, Quality of life

## Abstract

**Background:**

The study aimed to assess the frequency of malnutrition and its associated factors among patients with central nervous system inflammatory demyelinating diseases (CNSIDDs) and to explore their relationship with quality of life (QoL).

**Methods:**

A cross-sectional study at Siriraj Hospital included patients with CNSIDD aged > 18 years and matched healthy controls (HCs). Nutritional status and QoL were evaluated using the Mini Nutritional Assessment (MNA) and the 36-Item Short-Form Survey (SF-36), respectively. Spearman’s rank correlation was used to identify correlations between factors and SF-36 scores. Multivariate Poisson regression analyses utilized a combined “abnormal nutritional status” outcome (pooling “at-risk” and “malnourished” patients) to identify significant associated factors.

**Results:**

The study included 222 patients (100 multiple sclerosis (MS), 105 neuromyelitis optica spectrum disorder (NMOSD), and 17 myelin oligodendrocyte glycoprotein antibody-associated disease (MOGAD)) with a mean age of 46.2 years (standard deviation (SD) 15.2). Overall, 102/222 (45.9%) CNSIDD patients were at risk of or actively experiencing malnutrition. Mean MNA scores were 23.25 (SD 3.76) for MS, 22.84 (SD 3.50) for NMOSD, and 22.03 (SD 3.03) for MOGAD, compared to 26.07 (SD 1.88) for HC (P < 0.001). Malnutrition was found in 7.0% of MS and 8.6% of NMOSD patients, but 0% of MOGAD patients. However, the proportion of patients at risk of malnutrition was highest in the MOGAD group (70.6%), compared to MS (35.0%) and NMOSD (37.1%). SF-36 scores showed a significant positive correlation with MNA scores (r = 0.463, P < 0.001). Initial regression analysis indicated that a MOGAD diagnosis, high Expanded Disability Status Scale (EDSS) scores, and low body mass index were associated with abnormal nutritional status, although these associations remain subject to unmeasured treatment and comorbidity confounders.

**Conclusion:**

A significant proportion of CNSIDD patients face malnutrition or are at high risk, which correlates with poorer QoL and greater disease disability. Routine nutritional screening is an essential component of standard care for this population.

## Introduction

Central nervous system inflammatory demyelinating diseases (CNSIDDs) are characterized by immune-mediated damage to myelin, encompassing multiple sclerosis (MS), neuromyelitis optica spectrum disorder (NMOSD), and myelin oligodendrocyte glycoprotein antibody-associated disease (MOGAD) [1]. Depending on locations and extent of demyelinating lesions, the clinical symptoms could range from sensory disturbances to severe motor, visual, and cognitive impairments [2–4], which can significantly impact the quality of life (QoL) [5, 6].

Previous assessments using the Global Leadership Initiative on Malnutrition (GLIM) criteria found that approximately 20% of MS patients were classified as malnourished, while 80% were at risk [7]. Other MS cohorts report overlapping challenges: while 63.4% of patients were classified as overweight, malnutrition was simultaneously observed in up to 67.1% [8, 9]. Similarly, in NMOSD, nutritional status, assessed using the Mini Nutritional Assessment (MNA), is significantly worse than in healthy controls (HCs) [10]. Because malnutrition is independently associated with worsening disability in MS, addressing nutritional imbalances is imperative to optimize disease outcomes and enhance patients’ overall QoL [10, 11].

Thai populations possess distinct body habitus and dietary patterns compared to Western cohorts, with the general population often consuming fewer calories than globally recommended [12]. As a result, exploring the nutritional status of Thai CNSIDD has become increasingly important. Currently, no studies have examined malnutrition in this specific demographic, and data on the nutritional status of NMOSD and MOGAD patients remain particularly sparse. This study aimed to assess the prevalence of malnutrition in patients with MS, NMOSD, and MOGAD, evaluate health-related QoL, and identify clinical factors associated with nutritional decline.

## Materials and Methods

### Data collection and ethical considerations

This questionnaire-based cross-sectional study was conducted at Siriraj Hospital, Mahidol University. Patients were recruited from June 10, 2023, to February 22, 2025. The study protocol was first approved by the Siriraj Institutional Review Board Committee (COA No. Si 453/2023) on June 8, 2023 and was conducted in compliance with the Declaration of Helsinki.

### Participant selection

The inclusion criteria comprised patients aged over 18 years with MS, NMOSD (including both seropositive and seronegative AQP4 patients), or MOGAD. Patients were excluded if they lacked a definitive diagnosis or were unable to complete the nutritional and QoL questionnaires. To capture a representative, real-world clinical cohort, patients were not explicitly excluded based on concurrent medical or psychiatric comorbidities that are independently associated with malnutrition. Instead, these conditions were documented as baseline characteristics, and their potential confounding effects on nutritional status are addressed in the limitations.

For the HC group, we recruited 100 individuals at a 2:1 patient-to-control ratio. These controls were age-matched (within 5 years) and sex-matched to the patient cohort, and were recruited from hospital personnel and patient relatives. Exclusion criteria for controls included any history of neurological disorders, severe uncontrolled chronic illnesses, or conditions significantly impacting daily function or dietary habits.

### Nutritional status definitions

Nutritional status was defined and categorized using the full Mini Nutritional Assessment (MNA) questionnaire (maximum score of 30). Patients were classified into three standard categories based on their total MNA score: normal nutritional status (24.0–30.0 points), at risk of malnutrition (17.0–23.5 points), and malnourished (less than 17.0 points). Body mass index (BMI) was recorded as a baseline demographic variable and analyzed as an associated factor, but it was not used as the sole determinant of malnutrition.

### Clinical outcomes

The primary objective was to assess the prevalence of malnutrition among patients with MS, NMOSD, and MOGAD. Secondary objectives included: (1) comparing malnutrition rates and QoL across CNSIDD subtypes, and (2) identifying clinical factors associated with abnormal nutritional status.

### Statistical analysis

Frequencies are reported as percentages. The one-way analysis of variance (ANOVA) test was used to compare normally distributed continuous variables, and the Kruskal–Wallis test was applied for non-normally distributed variables. Categorical variables were analyzed using the chi-square test or Fisher’s exact test. Associations between parameters were evaluated using Spearman’s rank correlation.

“At risk” and “malnourished” MNA scores were pooled into a single “abnormal nutritional status” outcome for regression modeling. Univariate Poisson regression analysis with robust variance identified relevant factors (cutoff P < 0.10). Subsequently, variables meeting this threshold were entered into a backward stepwise multivariate Poisson regression model to determine significant independent factors associated with abnormal nutritional status (cutoff P < 0.05). Analyses were conducted using PASW Statistics version 26.0 and Stata BE version 19.5. A P-value < 0.05 was considered statistically significant.

## Results

### Demographic and baseline patient characteristics

We sequentially included 222 patients with an average age of 46.2 ± 15.2 years, of whom 188 (84.7%) were females. Nine patients (4.1%) had concurrent tumors or malignancies (two meningiomas, one acute myeloid leukemia, one diffuse large B-cell lymphoma, one myelodysplastic syndrome, one dermoid cyst, one myoma uteri, and two breast cancer), 11 (5.0%) had concurrent psychiatric diagnosis (six major depressive disorders, two psychosis, one bipolar disorder, one adjustment disorder, and one schizophrenia), and 54 (24.3%) patients had cardiometabolic risk factors. The HC group comprised 100 patients with an average age of 46.3 ± 3.2 years, of whom 85 (85.0%) were females. The mean age at disease onset was 35.7 ± 14.9 years. The median disease duration was 8.5 years (interquartile range (IQR): 3.9–14.5), and the median Expanded Disability Status Scale (EDSS) was 2.0 (IQR: 1.0–4.0). Demographic data are shown in Table 1.

**Table 1:** Demographic Data and Baseline Characteristics Among Patients With Central Nervous System Demyelinating Diseases

Characteristics	Healthy controls (n = 100)	Total (n = 222)	MS (n = 100)	NMOSD (n = 105)	MOGAD (n = 17)	P-value
Age at recruitment (years), mean ± SD	46.3 ± 3.3	46.2 ± 15.2	40.0 ± 12.8	52.5 ± 14.3	43.9 ± 18.2	< 0.001
Age of onset (years), mean ± SD	NA	35.7 ± 14.9	30.3 ± 11.8	40.7 ± 15.8	36.6 ± 14.9	< 0.001
Female sex, n (%)	85 (85.0)	188 (84.7)	76 (76.0)	101 (96.2)	11 (64.7)	< 0.001
BMI (kg/m^2^), mean ± SD	24.1 ± 4.9	23.5 ± 5.0	23.4 ± 5.0	23.4 ± 4.9	25.0 ± 6.1	0.448
Weight (kg), mean ± SD	62.4 ± 5.4	60.7 ± 14.2	62.2 ± 13.8	58.4 ± 13.0	65.6 ± 6.1	0.053
Number of attacks, median (IQR)	NA	3.0 (1.0–5.0)	2.0 (1.0–3.5)	3.0 (1.0–6.0)	3.0 (1.0–5.0)	0.039
ARR, median (IQR)	NA	0.4 (0.2–0.7)	0.4 (0.2–0.7)	0.4 (0.2–0.63)	0.6 (0.3–1.0)	0.164
Disease duration (years), median (IQR)	NA	8.5 (3.9–14.5)	6.7 (3.2–13.7)	10.9 (5.0–15.3)	5.7 (1.5–9.6)	0.017
Duration from last attacks (years), median (IQR)	NA	3.7 (1.7–7.9)	3.5 (1.8–7.9)	4.2 (1.9–8.2)	2.3 (1.7–6.3)	0.647
EDSS, median (IQR)	NA	2.0 (1.0–4.0)	1.0 (0.0–3.0)	3.0 (1.0–5.5)	1.0 (1.0–2.0)	0.01
No treatment, n (%)	NA	19 (8.6)	13 (13.0)	3 (2.9)	3 (17.6)	0.001
Prednisolone, n (%)	NA	0 (0.0)	0 (0.0)	0 (0.0)	2 (11.8)	0.006
Azathioprine, n (%)	NA	75 (33.8)	20 (20)	45 (42.9)	10 (58.8)	< 0.001
Mycophenolate mofetil, n (%)	NA	25 (11.3)	8 (8.0)	15 (14.3)	2 (11.8)	0.362
Rituximab, n (%)	NA	82 (36.9)	42 (42.0)	40 (38.1)	0 (0.0)	0.004
Fingolimod, n (%)	NA	9 (4.1)	9 (9.0)	0 (0.0)	0 (0.0)	0.003
Glatiramer acetate, n (%)	NA	1 (0.5)	1 (1.0)	0 (0.0)	0 (0.0)	0.542
Natalizumab, n (%)	NA	1 (0.5)	1 (1.0)	0 (0.0)	0 (0.0)	0.542
Satralizumab, n (%)	NA	2 (0.9)	0 (0.0)	2 (1.9)	0 (0.0)	0.325
Teriflunomide, n (%)	NA	4 (1.8)	4 (4.0)	0 (0.0)	0 (0.0)	0.083
Dimethyl fumarate, n (%)	NA	2 (0.9)	2 (2.0)	0 (0.0)	0 (0.0)	0.292

ARR: annualized relapsed rate; BMI: body mass index; EDSS: Expanded Disability Status Scale; IQR: interquartile range; MOGAD: myelin oligodendrocyte glycoprotein antibody-associated disease; MS: multiple sclerosis; NA: not available; NMOSD: neuromyelitis optica spectrum disorder; SD: standard deviation.

### MNA scores of patients with MS, NMOSD, and MOGAD compared with HC

The average MNA scores for all MS, NMOSD, and MOGAD patients were 22.96 ± 3.59. A total of 120/222 (54.05%) had normal nutritional status, 86/222 (38.74%) were at risk of malnutrition, and 16/222 (7.21%) were malnourished. The mean MNA scores were 23.25 ± 3.76 in MS, 22.84 ± 3.50 in NMOSD, and 22.03 ± 3.03 in MOGAD. The prevalence of malnutrition was 7/100 (7.0%), 9/105 (8.6%), and 0/17 (0%), and the risk of malnutrition was 35/100 (35.0%), 39/105 (37.1%), and 12/17 (70.6%) in MS, NMOSD, and MOGAD, respectively. However, the mean MNA score for the control population was significantly higher than that for the CNSIDD population (26.07 (SD 1.88); P < 0.001). The proportion of control participants with normal nutritional status was 89/100 (89.0%), and the risk of malnutrition was 11/100 (11.0%). There were differences in the proportions at risk of malnutrition and experiencing malnutrition, with the highest proportions in MOGAD patients (P = 0.019) and NMOSD patients (P = 0.020), respectively. Comparisons among the groups are shown in Figure 1.

**Figure 1:**
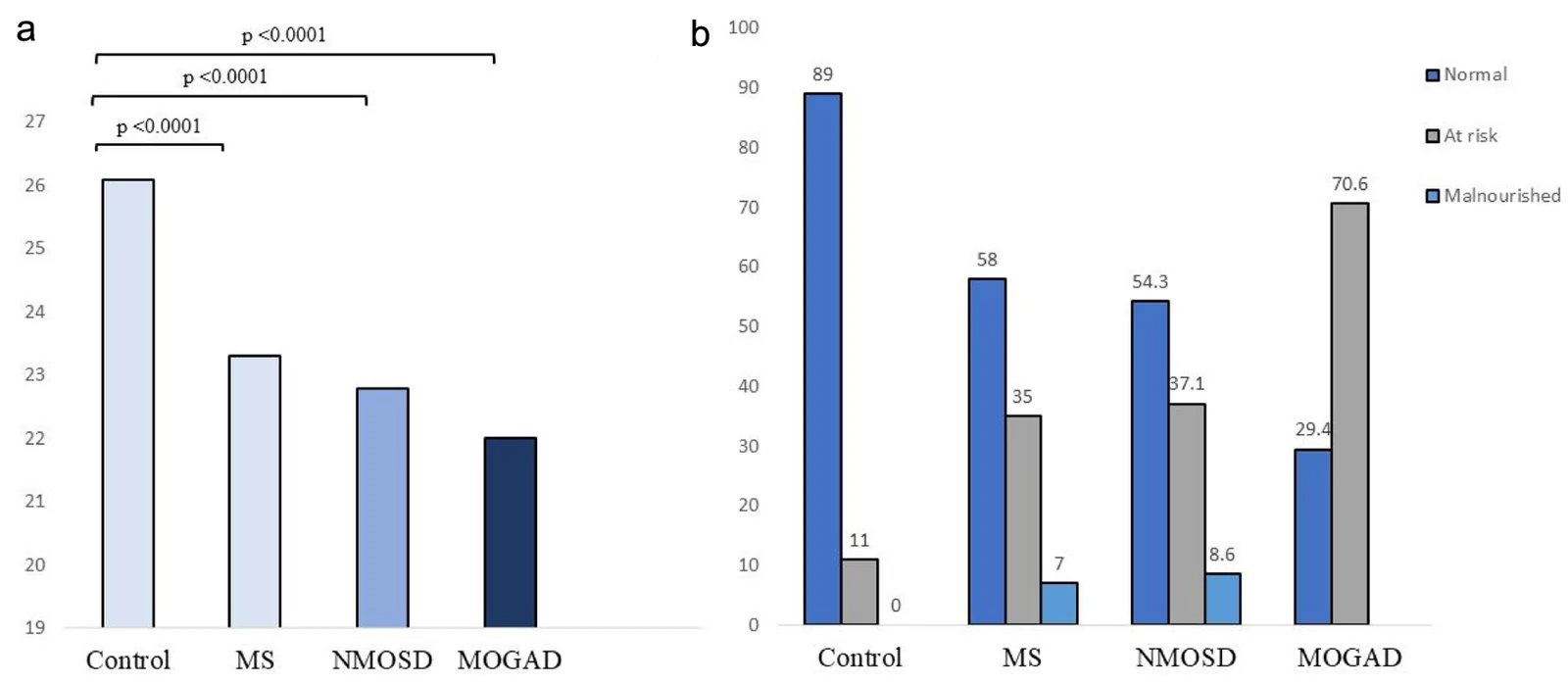
The nutritional status of patients is categorized by the MNA score. (a) Bar graph of the mean MNA score between each group. (b) Bar graph of the proportions of nutritional status of healthy controls, MS, NMOSD, and MOGAD patients. MNA: Mini Nutritional Assessment; MOGAD: myelin oligodendrocyte glycoprotein antibody-associated disease; MS: multiple sclerosis; NMOSD: neuromyelitis optica spectrum disorder.

### QoL status in CNSIDD patients

The overall 36-Item Short-Form Survey (SF-36) score was similar across groups, with a mean of 58.7 ± 15.5. There were no differences in the SF-36 subscores, including general health, physical function, physical role, bodily pain, vitality, social function, role emotion, and mental health. Full details of the SF-36 score were demonstrated in Supplementary Material 1 (jocmr.elmerjournals.com).

### Baseline and clinical factors associated with either at risk of malnutrition or malnutrition

The results demonstrated that patients with MOGAD had a significantly higher risk of abnormal nutritional status than MS patients (prevalence ratio (PR) = 2.02, 95% confidence interval (CI) 1.49–2.93). Additionally, higher EDSS scores (PR = 1.13, 95% CI 1.08–1.19) and lower BMI (PR = 1.09, 95% CI 1.06–1.14) were significantly associated with abnormal nutritional status. Furthermore, visual analysis was consistent with an inverse relationship between physical disability and nutritional status, with MNA scores declining as EDSS increased (Fig. 2; Table 2).

**Figure 2:**
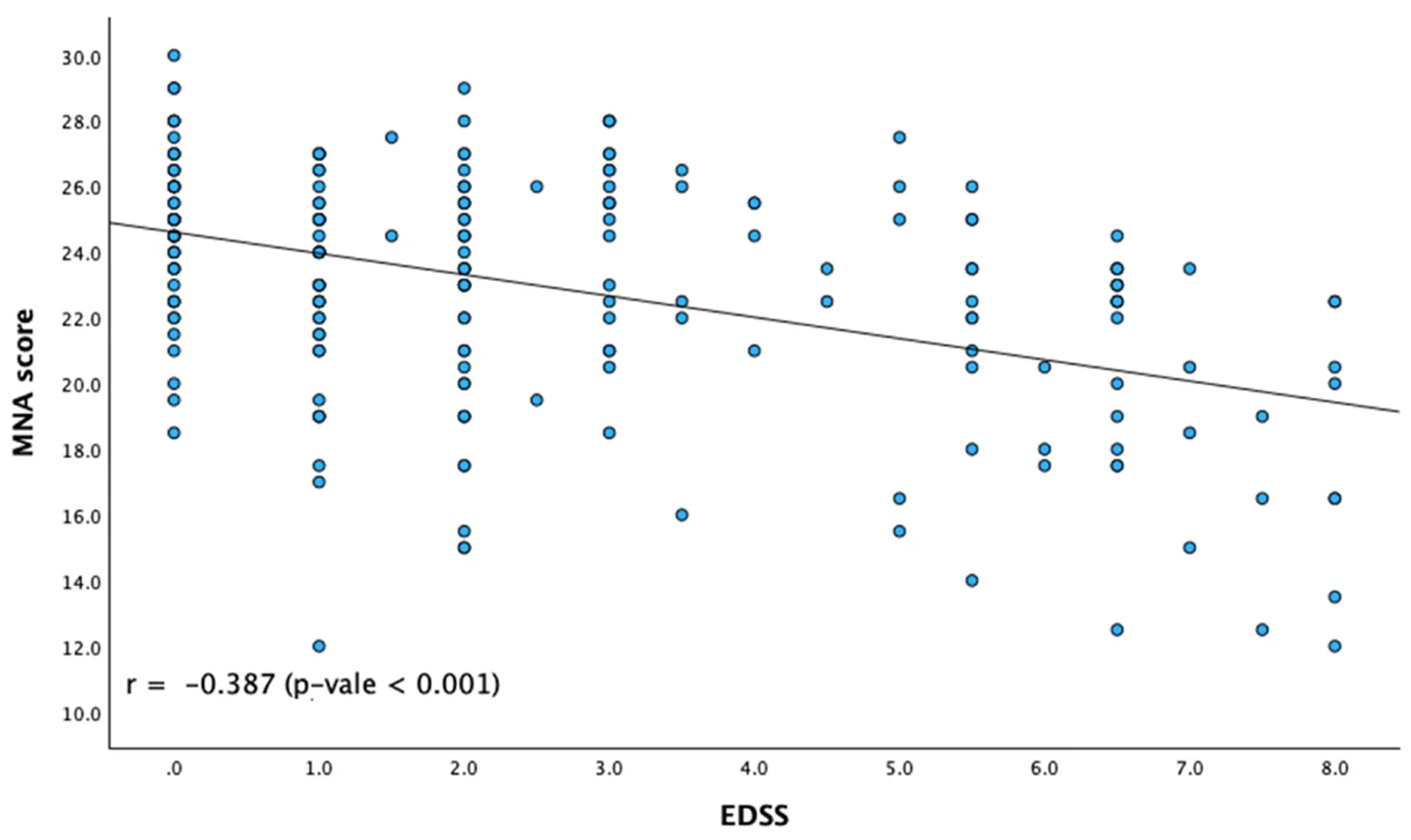
Correlation between clinical disability and nutritional status. The scatter plot demonstrates the relationship between EDSS scores and MNA scores across the demyelinating disease cohorts. EDSS: Expanded Disability Status Scale; MNA: Mini Nutritional Assessment.

**Table 2:** Univariate and Multivariate Poisson Regression to Predict the Risk of Malnutrition and Patients at Risk of Malnutrition

	Univariate regression		Multivariate regression	
	Crude PR 95%CI	P-value	Adjusted PR (95% CI)	P-value
Diagnosis				
MS	Ref	Ref	Ref	Ref
NMOSD	1.09 (0.80–1.48)	0.594	0.94 (0.72–1.23)	0.635
MOGAD	1.68 (1.15–2.47)	0.008	2.02 (1.49–2.93)	< 0.001
Sex				
Male	Ref	Ref		
Female	1.24 (0.79–1.95)	0.357		
Concurrent tumors/malignancy	1.22 (0.67–2.23)	0.519		
Concurrent psychiatric disorders	1.63 (1.10–2.42)	0.014	NA	NA
Preexisting cardiometabolic risk factors	1.03 (0.77–1.37)	0.859		
Number of attacks	1.04 (1.01–1.05)	< 0.001	1.01 (0.99–1.02)	0.228
Age	1.01 (0.97–1.02)	0.206		
Disease duration	1.02 (1.00–1.03)	0.064	1.01 (0.99–1.02)	0.377
Lower BMI (continuous)	1.11 (1.08–1.14)	< 0.001	1.09 (1.06–1.14)	< 0.001
EDSS	1.16 (1.11–1.21)	< 0.001	1.13 (1.08–1.19)	< 0.001
Treatment categories				
No treatment	Ref	Ref		
Low-moderate efficacy DMT	1.18 (0.66–2.11)	0.584		
High efficacy DMT	1.30 (0.72–2.33)	0.385		

Adjusted PRs were calculated using a stepwise multivariate model adjusting for diagnosis subtype, number of attacks, disease duration, BMI, and EDSS scores. Variables with a univariate P value < 0.10 that did not remain significant in the final model following stepwise selection are designated as not applicable (NA). BMI: body mass index; CI: confidence interval; DMT: disease-modifying therapy; EDSS: Expanded Disability Status Scale; MOGAD: myelin oligodendrocyte glycoprotein antibody-associated disease; MS: multiple sclerosis; NMOSD: neuromyelitis optica spectrum disorder; PR: prevalence ratio.

## Discussion

This study evaluated the nutritional status of patients with MS, NMOSD, and MOGAD. Nearly 46% of CNSIDD patients were at risk of or diagnosed with malnutrition compared to 11% of HCs. MNA scores were significantly lower in CNSIDD patients than in HCs. SF-36 scores also showed significant correlations with MNA scores suggesting that lower nutritional status tracks with poorer QoL with nutritional status. Initial regression analysis suggested that a MOGAD diagnosis, high EDSS, and low BMI were associated with abnormal nutritional status; however, these findings must be interpreted with caution, as the model did not fully control for treatment-related confounders or comorbidity burden.

In this cohort, the disease group had a higher proportion of patients with at-risk malnutrition and malnutrition than the control group. The prevalence of nutritional risk in our cohort (45.9%) is notably lower than the 67.1% reported in Egyptian MS patients [9] and the 87.8% reported in Turkish cohorts [8, 13, 14]. The rate of NMOSD is similarly high, at approximately 85% [10]. Several factors may explain this variance. First, the assessment tools differed; while we utilized the MNA, other studies employed the GLIM [14] or Subjective Global Assessment (SGA) [9], which may have different sensitivity thresholds. Second, our cohort consisted entirely of outpatients with a relatively low median EDSS of 2.0. In contrast, studies reporting higher rates often included hospitalized patients or those with more advanced disability, where dysphagia and limited mobility significantly exacerbate nutritional decline. Finally, dietary habits and the lower average BMI in the general Thai population compared to Western or Middle Eastern cohorts may influence the baseline captured by the MNA.

Our study revealed that NMOSD patients have the highest proportion of malnutrition status. The findings could be due to their more severe clinical presentation, both of which are associated with a higher risk of malnutrition [15, 16]. Similarly, MOGAD patients exhibited the highest proportion of “at-risk” malnutrition. We hypothesize that this may be related to shorter durations since their last attack in our cohort and differing acute management strategies.

Improved nutritional status was associated with lower disability levels and higher QoL in our cohort, while some evidence suggests that dietary interventions might influence symptom severity [17]. For MS patients, neurodegeneration could be present even in the early stage of the disease, which could lead to long-term disability and worsening of the disease [18]. Therefore, implementing strategies to mitigate neurodegeneration is essential. One approach involves dietary control, which may help reduce oxidative stress and protect against chronic demyelination [19–21]. Furthermore, maintaining an optimal diet can alleviate fatigue and enhance patients’ QoL [20]. Previous studies have suggested that adopting a Mediterranean diet is beneficial for individuals with MS [22, 23]. Regarding micronutrients, MS and NMOSD patients often have vitamin D deficiency, which could result in further disease progression and disability [24–26]. Nevertheless, conflicting data still exist regarding the role of vitamin D supplementation in MS patients [27].

Given the study’s significant findings, implementing a health policy to address nutritional disorders is crucial. The 2017 ESPEN Guideline on Clinical Nutrition in Neurology recommends screening for malnutrition at the time of MS diagnosis. This includes screening for dysphagia in high-risk groups, such as patients with severe disabilities, cerebellar dysfunction, or prolonged disease duration. However, standard screening tools for these patients have not yet been recommended [28]. Therefore, adapting nutritional intake recommendations to the Thai population’s diet and implementing methods to improve compliance could potentially benefit Thai CNSIDD patients [29].

The nutritional deficits could reflect systemic involvement described across chronic immune-mediated diseases. Systemic manifestations have similarly been reported in other organ autoimmune diseases, including gastrointestinal disorders such as celiac disease [30] and diseases affecting other primary target organs, such as psoriasis [31] and Hashimoto thyroiditis [32]. Therefore, inflammatory pathways, instead of a demyelination-specific process, may also contribute to nutritional decline across chronic autoimmune conditions.

Our study has several limitations. First, the cross-sectional design and small sample sizes within subgroups (particularly the 17 MOGAD patients) limit generalizability and prevent the establishment of strict causation. Second, our multivariable models were not adjusted for several clinically important determinants of nutritional status. Specifically, patients with conditions independently associated with malnutrition, such as concurrent malignancies or psychiatric disorders, were included without appropriate statistical adjustment. Furthermore, the majority of patients had histories of varied immunotherapies. We did not adjust the regression model for cumulative corticosteroid exposure or duration of steroid therapy, as our registry only captured a binary metric of current use at the time of the questionnaire. This is a significant potential confounder, given the systemic glucocorticoid requirements in MOGAD patients.. As dose and duration data were not available for any subgroup, a corresponding sensitivity analysis could not be performed; prospective collection of cumulative glucocorticoid exposure is recommended. Additionally, dietary habits and the lower average BMI in the general Thai population compared to Western or Middle Eastern cohorts may influence the baseline captured by the MNA, which was developed and validated primarily in elderly Western populations. Therefore, the lower baseline MNA scores observed in our study may partially reflect the baseline population rather than pathological malnutrition alone. Finally, while highly validated, the MNA lacks comprehensive dietary tracking, which may have led to the oversight of specific micronutrient deficiencies.

In conclusion, this study explicitly highlighted the risk and evidence of malnutrition in patients with MS, NMOSD, and MOGAD. However, the overall MNA scores did not reveal significant differences between patient subtypes. The alarming prevalence of malnutrition risk necessitates targeted interventions. Regular monitoring and individualized nutritional support are essential components of comprehensive care for individuals with demyelinating diseases to potentially improve their health outcomes and QoL. Future studies should explore the underlying causes of nutritional deficiencies in these populations and develop customized diet strategies to mitigate these risks.

## Supplementary Material

Suppl 1 The 36-Item Short-Form Survey comparing health-related quality of life in NMOSD, MS, and MOGAD.

## Data Availability

The data supporting the findings of this study are available from the corresponding author (Dr. Jiraporn Jitprapaikulsan) upon reasonable request..

## Abbreviations

AQP4: aquaporin-4
ARR: annualized relapsed rate
BMI: body mass index
CI: confidence interval
CNSIDD: central nervous system inflammatory demyelinating diseases
DMT: disease-modifying therapy
EDSS: Expanded Disability Status Scale
ESPEN: European Society for Clinical Nutrition and Metabolism
GLIM: Global Leadership Initiative on Malnutrition
HC: healthy controls
IQR: interquartile range
MNA: Mini Nutritional Assessment
MOGAD: myelin oligodendrocyte glycoprotein antibody-associated disease
MS: multiple sclerosis
NA: not available
NMOSD: neuromyelitis optica spectrum disorder
PR: prevalence ratio
QoL: quality of life
SD: standard deviation
SF-36: 36-Item Short Form Survey
SGA: Subjective Global Assessment
