# Supplementary material for: Nutritional Status in Multiple Sclerosis, Neuromyelitis Optica Spectrum Disorder, and Myelin Oligodendrocyte Glycoprotein Antibody-Associated Disease
Source: J Clin Med Res. 2026 Sep 26;18(9):679–86. doi: 10.14740/jocmr6676 (PMC13661087; doi:10.14740/jocmr6676)
Supplement: Supplementary file 1 [file jocmr-18-09-679-s001.docx]

**Suppl 1.** The 36-Item Short-Form Survey Comparing Health-Related Quality of Life in NMOSD, MS, and MOGAD

| Outcome | Total  n = 222 | MS  n = 100 | NMOSD  n = 105 | MOGAD  n = 17 |
| --- | --- | --- | --- | --- |
| Total SF-36, mean [SD] | 58.67  [15.54] | 58.90 [15.54] | 58.24 [15.65] | 60.10 [15.67] |
| SF-36 general health, mean [SD] | 49.69  [21.48] | 49.11 [21.69] | 49.31 [21.86] | 55.35 [17.91] |
| SF-36 physical function, mean [SD] | 67.52  [32.97] | 69.20 [32.78] | 63.93 [33.83] | 79.41 [26.21] |
| SF-36 role physical, mean [SD] | 34.47  [21.07] | 36.00 [19.78] | 32.36 [22.12] | 38.24 [21.86] |
| SF-36 Bodily pain, mean [SD] | 64.68  [25.41] | 68.28 [24.75] | 59.96 [25.75] | 72.88 [22.79] |
| SF-36 Vitality, mean [SD] | 60.48  [18.04] | 60.00 [16.04] | 61.44 [18.57] | 57.35 [25.26] |
| SF-36 Social function, mean [SD] | 84.62  [21.64] | 83.50 [21.46] | 86.90 [20.42] | 77.21 [28.38] |
| SF-36 Role emotion, mean [SD] | 39.47  [18.68] | 39.83 [18.02] | 39.42 [19.02] | 37.50 [21.52] |
| SF-36 mental health, mean [SD] | 67.38  [18.05] | 66.16 [18.54] | 69.54 [16.49] | 61.18 [21.85] |

Abbreviation: NMOSD, Neuromyelitis optica spectrum disorder; MS, Multiple sclerosis; MOGAD, Myelin Oligodendrocyte Glycoprotein Antibody Disease; SD, Standard Deviation, SF-36; 36-item short-form survey.
